# Population genomics reveals gene flow and positive selection patterns in the wine-related yeast *Hanseniaspora uvarum*

**DOI:** 10.1007/s44154-026-00319-z

**Published:** 2026-07-27

**Authors:** Ruiqi Ma, Hui Wang, Yue Wei, Yue Sun, Jie Xue, Yi Qin, Shiheng Tao, Yanlin Liu

**Affiliations:** 1 College of Life Sciences, Bioinformatics Center, and State Key Laboratory for Crop Stress Resistance and High-Efficiency Production, Northwest A&F University, Yangling, Shaanxi 712100 China; 2 College of Enology, Northwest A&F University, Yangling, Shaanxi 712100 China; 3 Xinjiang Research Institute of Agriculture in Arid Areas, Northwest A&F University, Urumqi, Xinjiang 830091 China; 4 School of Enology and Horticulture, Ningxia University, Yinchuan, Ningxia 750021 China; 5 Engineering Research Center of Grape and Wine, Ministry of Education, Yinchuan, Ningxia 750021 China; 6 China National Research Institute of Food & Fermentation Industries, Beijing, 100015 China

**Keywords:** *Hanseniaspora uvarum*, Non-*Saccharomyces* yeasts, Population genomics, Gene flow, Positive selection, Pangenome

## Abstract

*Hanseniaspora uvarum* is a representative non-*Saccharomyces* species that plays a significant role in fermentation processes such as winemaking. In recent years, this species has gained attention in food engineering and evolutionary biology. However, the population genomic signatures in this species remain poorly understood. In this study, a population genomics analysis was conducted on 151 *H. uvarum* strains (45 from Ningxia, China; 21 from other regions of China; 67 from Australia; and 18 from other regions or of unspecified origin), and a pangenome analysis was performed on 159 strains, incorporating eight additional genome assemblies. Phylogenetic analysis, ancestry coefficient analysis, and principal component analysis generally distinguished Chinese strains from those sampled on other continents. However, substantial post-divergence gene flow and introgression were inferred between intercontinentally paired clades. Positively selected candidate genes exhibited region-specific patterns: GO terms related to the positive regulation of filamentous growth in response to external stimuli were significantly enriched in the Ningxia strains; the stress-related GO term “cytoplasmic stress granule” was significantly enriched in both the Ningxia and Australian strains, but with distinct sets of associated genes. Although the samples were primarily isolated from anthropogenic environments, *H. uvarum* exhibited an open pangenome, indicating substantial adaptive potential to diverse stresses. This study advances our understanding of the evolutionary dynamics of *H. uvarum* and establishes a genomic foundation for future ecological and industrial research on this yeast.

**Supplementary Information:**

The online version contains supplementary material available at 10.1007/s44154-026-00319-z.

## Introduction

*Hanseniaspora uvarum*, a non-*Saccharomyces* species commonly isolated from grapes and spontaneous wine fermentations (Drumonde-Neves et al. 2021), exhibits high abundance and isolation frequency during the early stages of spontaneous wine fermentation. However, the abundance of *H. uvarum* decreases as ethanol concentration increases in the later stages (Sternes et al. 2017; Feng et al. 2020). During wine fermentation, inoculation with *H. uvarum* can increase the content of esters and volatile phenols, reduce volatile acidity, and improve the sensory characteristics of wine (Tristezza et al. 2016; Hu et al. 2018; Wang et al. 2024). In addition, *H. uvarum* contributes to various fermentation processes and agricultural applications beyond wine production. It is not only found in fruits and their spontaneous fermentations, including those of oranges, apples, and pineapples (Arias et al. 2002; Valles et al. 2007; Dellacassa et al. 2017), but also involved in the fermentation of cocoa beans and coffee (Pereira et al. 2016; Figueroa-Hernández et al. 2019), and additionally serves as a biocontrol agent (Tian et al. 2021).

In recent years, *H. uvarum* has attracted growing research interest not only in agriculture and food engineering (Sun et al. 2025; Yang et al. 2025), but also in phylogenetic biogeography, evolutionary biology, and genomics (Albertin et al. 2016; Steenwyk et al. 2019; Saubin et al. 2020; Haase et al. 2024; Onetto et al. 2025). *Hanseniaspora* is divided into two evolutionary lineages: the Faster-Evolving Lineage (FEL) and the Slower-Evolving Lineage (SEL); *H. uvarum* belongs to the FEL. Compared with the SEL, the FEL species exhibit a lower guanine–cytosine (GC) content and smaller genome sizes. The stem lineages of both FEL and SEL exhibited accelerated sequence evolution, with the evolutionary rate of the SEL stem lineage being lower than that of the FEL stem lineage. However, the crown lineages of both clades showed reduced evolutionary rates. Both lineages of *Hanseniaspora* have lost some genes related to the cell cycle and DNA repair, and the loss in the FEL was more severe (Steenwyk et al. 2019). Additionally, a novel cis-regulatory mode of core histones emerged simultaneously with gene loss in *H. uvarum* (Haase et al. 2024).

The aforementioned studies have preliminarily revealed certain biological characteristics of *H. uvarum*, providing a foundation for deeper investigation into its evolutionary patterns. However, compared with *S. cerevisiae*, which has been well characterized in population genomics and pangenomics (Strope et al. 2015; Duan et al. 2018; Peter et al. 2018; Lee et al. 2022), relevant studies and knowledge of *H. uvarum* remain considerably underexplored. As one of the world's major grape-producing countries, China represents a rich source of *H. uvarum* strains for population-level studies. However, the evolutionary characteristics of wine-associated *H. uvarum* strains from China remain poorly understood. In this study, we performed whole-genome sequencing (WGS) of 65 wine-related *H. uvarum* strains isolated from major wine-producing regions in northwestern China. An additional 86 publicly available sequencing datasets were retrieved and integrated with the newly generated sequences. These combined data were used to investigate the population genomics of wine-related *H. uvarum* across multiple continents, primarily from samples originating in China and Australia. Finally, the pangenome of *H. uvarum* was constructed. Overall, this study deepens our understanding of the evolutionary patterns of *H. uvarum* and lays a foundation for future ecological and industrial research on this species.

## Results

### Genome-wide variation statistics

Sixty-five *H. uvarum* strains were collected from northwest China (Supplementary Fig. S1 and Table S1). The nuclear DNA sequencing depth of these strains ranged from 725.56 × to 1,543.38 ×, with a median of 1,197.04 ×; for the 86 publicly available strains, sequencing depths ranged from 53.95 × to 267.91 ×, with a median of 121.51 × (Supplementary Table S1). Through the combined use of EDTA (RRID: SCR_022063, v2.0.1) (Ou et al. 2019) and RepeatMasker (RRID: SCR_012954, v4.1.5), 145,357 bp of repetitive and low-complexity sequences were identified in the reference genome, accounting for approximately 1.64% of its total length. After filtering out variants in these regions, 575,222 high-quality variants were called across 151 strains, including 512,977 SNPs, 16,207 insertions, 17,070 deletions, 14,439 complex insertions and deletions (InDels), and 14,529 mixed loci that could not be classified as either SNPs or InDels. Downstream analyses were performed on 483,564 biallelic SNPs.

### Phylogenetic relationships among *H. uvarum *isolates

**Fig. 1:**
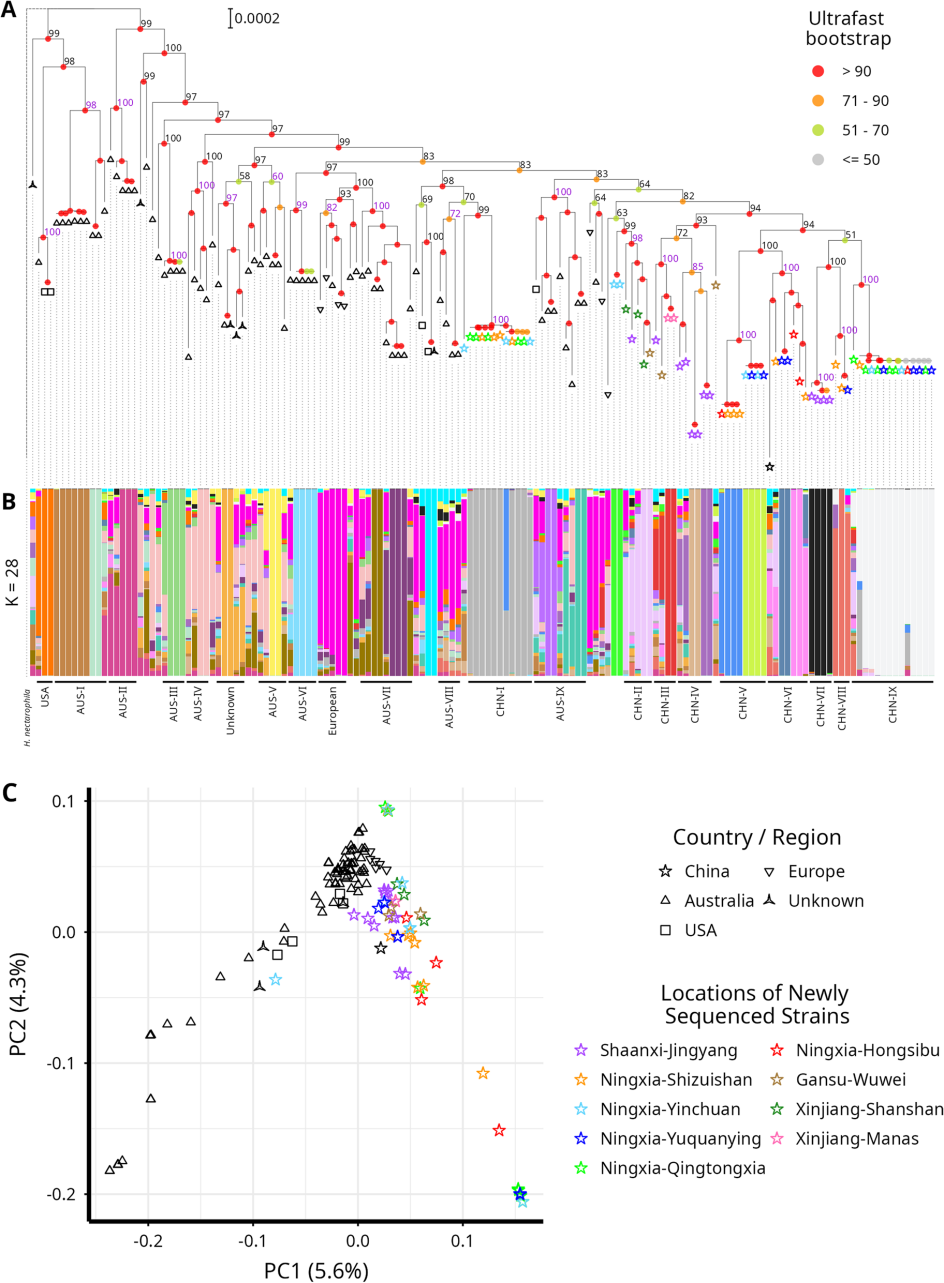
Phylogenetic tree, ancestry coefficient analysis, and principal component analysis (PCA) of 151 *H. uvarum* strains. **A** The ML tree of *H. uvarum*, with *H. nectarophila* designated as the outgroup. Ultrafast bootstrap support values are shown at nodes as numeric labels and colored circles. Values for the 21 delimited clades are indicated in violet numerals. Tip symbols denote sampling locations, as defined in Fig. 1C. **B** Ancestry coefficient analysis using sNMF at *K* = 28. **C** PCA scatter plot of PC1 versus PC2

Phylogenetic trees were inferred using two independent and complementary analytical frameworks: (1) A maximum likelihood (ML) tree was constructed from the concatenated CDS alignment, with *Hanseniaspora nectarophila* designated as the outgroup (Fig. 1A; Supplementary Fig. S2); (2) A dissimilarity matrix was calculated based on whole-genome SNPs. A BioNJ tree was then constructed from this matrix and rooted using the midpoint rooting method (Supplementary Fig. S2). Clades were delimited based on phylogenetic evidence from both the ML and BioNJ tree topologies, as well as on geographic sampling information. The ML and BioNJ trees yielded largely consistent clade assignments. In the ML tree, 129 of the 151 strains (85.4%) were assigned to 21 clades. Among these 129 strains, only five exhibited inconsistent clade assignments between the two trees (Supplementary Fig. S2; Supplementary Note). The CHN-I, CHN-V, CHN-VI, CHN-VIII, and CHN-IX clades consisted predominantly of strains sampled in Ningxia. The CHN-IV and CHN-VII clades consisted exclusively of strains sampled in Jingyang County, Shaanxi Province. Phylogenetic analysis broadly distinguished strains sampled in China from those sampled on other continents. In the ML tree, clades CHN-II through CHN-IX, together with four strains not assigned to any named clade (three from China and one from Europe), formed a monophyletic group.

Ancestry coefficient analysis (Fig. 1B, Supplementary Figs. S3–S5) was conducted using sNMF (v1.2) (Frichot et al. 2014) on 85,149 pruned biallelic SNPs. The cross-entropy value reached its minimum at *K* = 28 (Supplementary Fig. S5), and strains from the same clade shared similar ancestry coefficients. Principal component analysis (PCA) of the genetic data revealed that the first two principal components broadly distinguished Chinese strains from those sampled on other continents (Fig. 1C) and cumulatively explained 9.87% of the total variance (Supplementary Fig. S6).

Prior to calculating population genetic parameters, clonal strains were excluded. The average nucleotide diversity (π) of Chinese wine-related strains was 0.00780, and that of Australian wine-related strains was 0.00784, values that were nearly identical. The average nucleotide divergence (*d*_*XY*_) between the two regional populations was 0.00832. Hudson's *F*_ST_ (Hudson et al. 1992) between the two populations was 0.03376, indicating relatively low genetic differentiation.

### Gene flow and introgression between paired clades

**Fig. 2:**
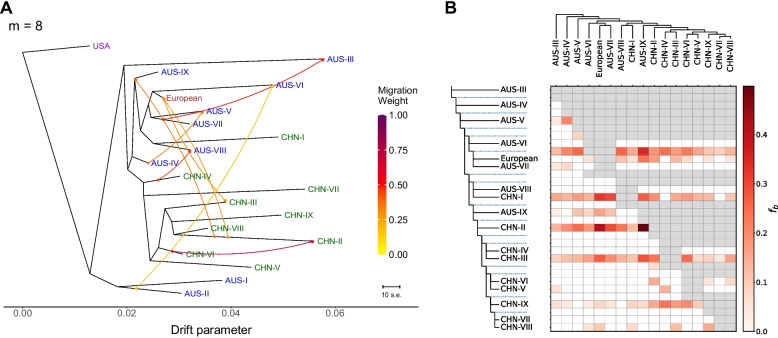
Detection of gene flow and introgression. **A** Detection of gene flow between paired clades using TreeMix, with the number of migration edges (*m*) set to 8. **B** The *f*-branch statistics quantifies excess sharing of derived alleles between the branch on the *y*-axis (relative to its sister branch) and the clades on the *x*-axis. The population trees shown on the *x*- and *y*-axes are constructed based on Fig. 1A

Gene flow between paired clades was modeled using TreeMix (v1.13) (Pickrell and Pritchard 2012) (Fig. 2A; Supplementary Fig. S7). The optimal number of migration edges (*m*) was determined by fitting four parametric models (piecewise linear, bent cable, simple exponential, and non-linear least squares) to the log-likelihood curve (Supplementary Fig. S8A). At *m* = 8, all four parametric models had reached or surpassed their respective change points, and the TreeMix model explained 95.7% of the variance in relatedness between clades (Supplementary Fig. S8B). Under this optimal TreeMix model (Fig. 2A), clades CHN-II through CHN-IX formed a cluster. Intercontinental gene flow was inferred (Fig. 2A) from the CHN-IV clade to the AUS-VIII clade, from the CHN-II clade to the European clade, and from the European clade to the CHN-III clade. Notably, the CHN-II and European clades clustered together in the BioNJ tree (Supplementary Fig. S2). Analysis at additional *m* values (Supplementary Fig. S7) revealed intercontinental gene flow from multiple Australian clades to multiple Chinese clades at *m* ≥ 10, with the exception of *m* = 12 or 13; from the USA clade to the CHN-VIII clade at *m* = 10, 11, 14, and 15; from the CHN-IV clade to the USA clade at *m* = 11 and 12; and from the AUS-VIII clade to the USA clade and from the USA clade to the AUS-IX clade, both at m ≥ 13.

The *f*-branch (*f*_*b*_) statistic, an extension of the *f*4-ratio, identifies excess allele sharing between paired clades as a signature of introgression (Malinsky et al. 2018, 2021). The *f*_*b*_ statistic indicated excess allele sharing between paired clades from Europe, Australia, and China (Fig. 2B). The highest intercontinental *f*_*b*_ value (49.84%) was observed between the CHN-II and AUS-IX clades, suggesting introgression between them. This introgression signature was consistent with the gene flow from the CHN-II clade to the AUS-IX clade inferred by the TreeMix model at *m* = 14 (Supplementary Fig. S7). Relatively high *f*_*b*_ values were observed between the CHN-II and European clades (41.20%), between the CHN-III and European clades (26.87%), and between the CHN-III and AUS-IX clades (28.28%). These values were consistent with gene flow from the CHN-II clade to the European clade and from the European clade to the CHN-III clade inferred by the optimal TreeMix model (*m* = 8; Fig. 2A), as well as with gene flow from the AUS-IX clade to the CHN-III clade inferred by the TreeMix model at *m* = 10 (Supplementary Fig. S7). Additional relatively high *f*_*b*_ values were observed between the CHN-I and European clades (32.33%) and between the European and AUS-IX clades (23.24%). Taken together, these results indicated that *H. uvarum* exhibited a widespread pattern of intercontinental gene flow and introgression, reflecting complex dispersal dynamics across continents.

### Positive selection analysis

**Fig. 3:**
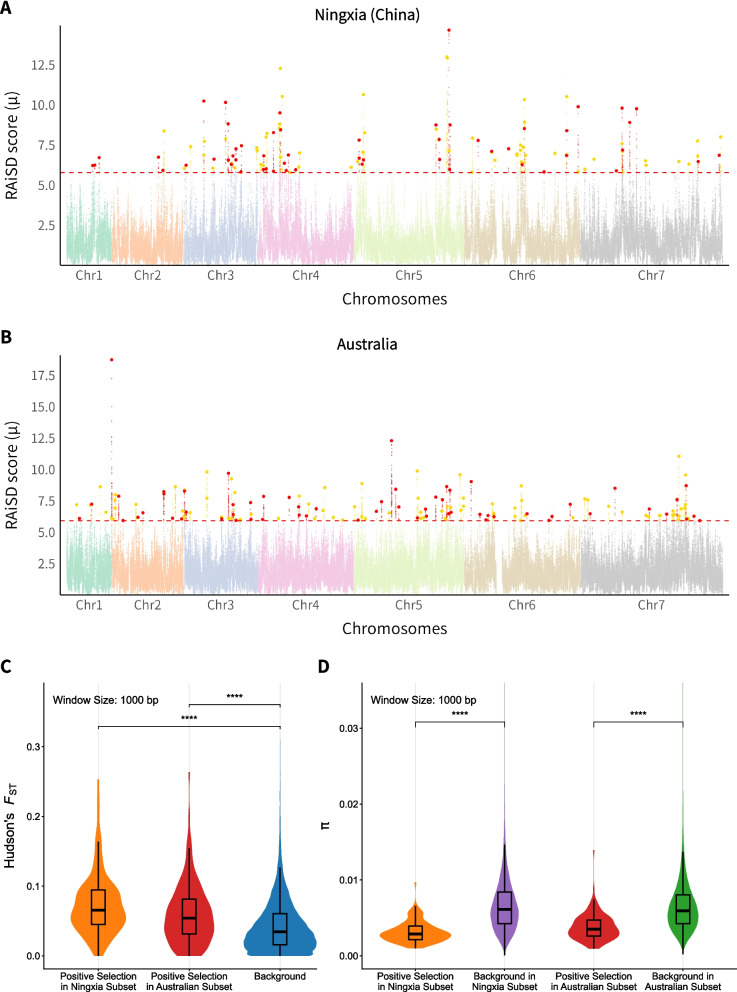
Identification of candidate genes under positive selection. **A**, **B** RAiSD analysis revealed candidate genes under positive selection in the Ningxia (China) subset and in the Australian subset. The horizontal red line indicates the 99.5th percentile threshold. Red dots represent genes associated with significantly enriched GO and KEGG terms, and yellow dots represent genes that are not involved in these terms. Larger dots represent the highest RAiSD score for the CDS region(s) of each gene. **C**, **D** Comparison of *F*_ST_ and genetic diversity (π) between the CDS of candidate genes under positive selection and the background (overall CDS across the genome). Asterisks designate significant differences between candidate positively selected regions and the background, as determined by the Wilcoxon rank-sum test (^ns^ Not significant, with *p* > 0.05; * *p* ≤ 0.05; ** *p* ≤ 0.01; *** *p* ≤ 0.001; **** *p* ≤ 0.0001)

For a subset of strains collected from Ningxia (China) and a subset of Australian strains (Supplementary Table S1), RAiSD (v2.9) (Alachiotis and Pavlidis 2018) was used to identify genes under positive selection. Both subsets excluded clonal strains. Additionally, strains from outside Ningxia were excluded from the analysis because strain collection across China was unevenly distributed between Ningxia and other regions, potentially introducing bias into allele frequency estimates. These analyses, conducted separately for each subset, revealed that loci with the top 0.5% of RAiSD scores overlapped with the CDS regions of 117 genes in the Ningxia subset and 141 genes in the Australian subset (Fig. 3A–B; Supplementary Tables S2 and S3). These genes were inferred to be candidates under positive selection, with 23 shared between the two subsets. Compared with genome-wide CDS background levels, Hudson's *F*_ST_ values in genomic windows containing RAiSD outliers were significantly higher, and the corresponding nucleotide diversity (π) values were significantly lower (Fig. 3C–D).

**Fig. 4:**
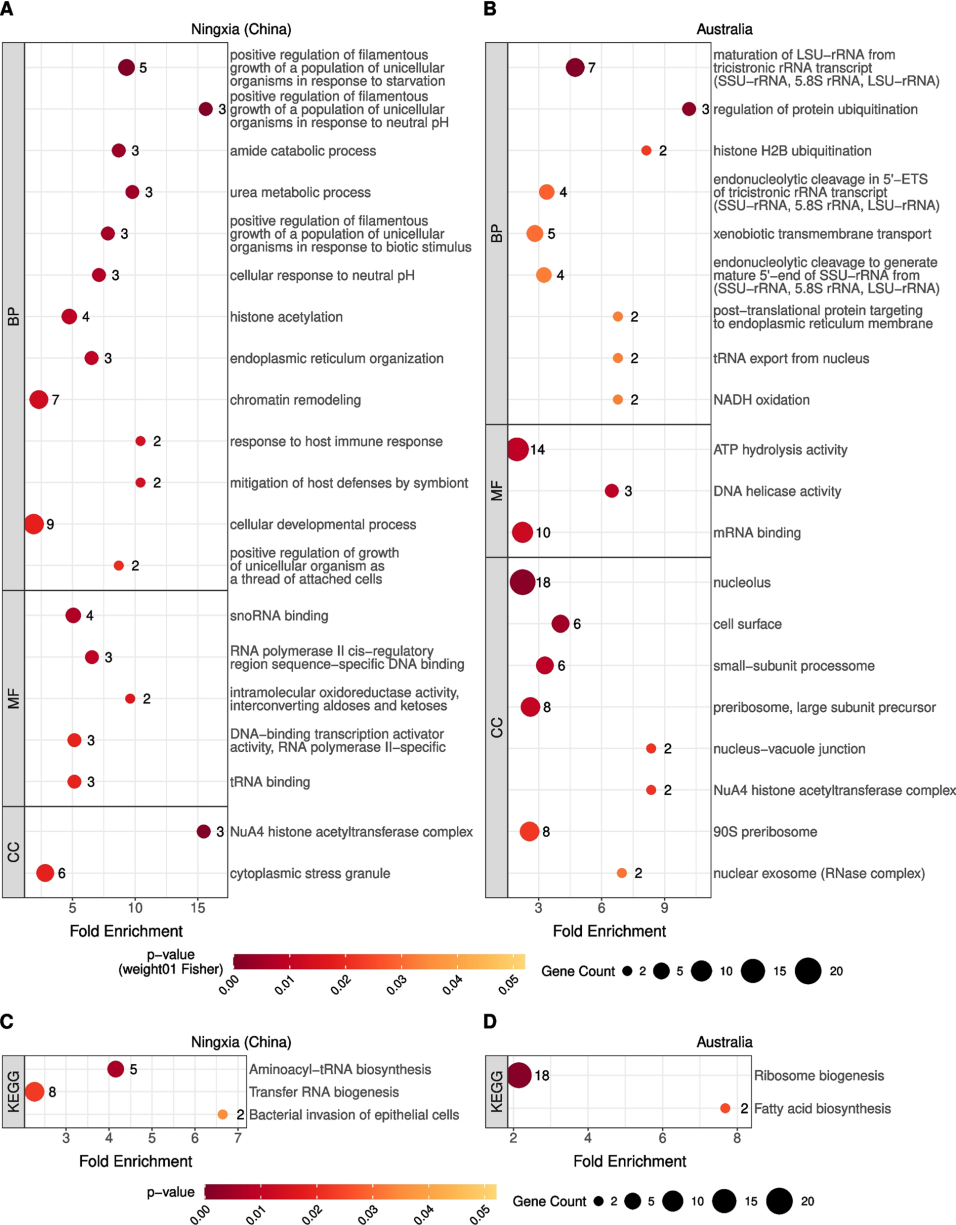
GO and KEGG enrichment analyses of candidate genes under positive selection in the Ningxia (China) subset and in the Australian subset. The numbers to the right of the dots represent the number of genes associated with the corresponding terms. **A**, **B** The top 20 significant GO terms with *p* ≤ 0.05 for both the weight01 and classic Fisher methods. See Tables S4 and S5 for additional information. **C**, **D** Significant enrichment of KEGG pathways. See Tables S6 and S7 for additional information. BP: Biological Process; MF: Molecular Function; CC: Cellular Component; KEGG: Kyoto Encyclopedia of Genes and Genomes

Gene Ontology (GO, RRID: SCR_002811) and Kyoto Encyclopedia of Genes and Genomes (KEGG, RRID: SCR_012773) pathway enrichment analyses were performed on the candidate genes under positive selection. In the Ningxia subset, 44 GO terms (*p* ≤ 0.05 in both the classic and weight01-weighted Fisher’s exact tests) and 3 KEGG pathways (*p* ≤ 0.05) were significantly enriched (Fig. 4A and C; Supplementary Tables S4 and S6). These terms and pathways were primarily associated with stress responses, metabolic processes, RNA processing, translation, chromatin remodeling, and histone acetylation, among others. Among the stress-related GO terms, four are direct child terms of “positive regulation of filamentous growth of a population of unicellular organisms.” These child terms correspond to the regulation of filamentous growth under conditions of starvation, pH stress, and biotic stimulus, as well as “positive regulation of growth of unicellular organism as a thread of attached cells.” These four enriched terms collectively encompass six associated genes. Of these six genes, two are also annotated to the significantly enriched GO term “phenotypic switching.” Other stress-related terms included “response to host immune response” and “cytoplasmic stress granule” (granules that appear in the cytoplasm under cellular stress). Metabolism-related terms included nitrogen metabolism (amide catabolism, urea metabolism, pyrimidine nucleobase biosynthesis) and carbon metabolism (pentose-phosphate shunt, glyceraldehyde-3-phosphate metabolism, interconversion of aldoses and ketoses). RNA-related terms and pathways included tRNA binding, tRNA aminoacylation, tRNA biogenesis, and snoRNA binding. The significantly enriched GO terms and KEGG pathways also included RNA polymerase II-dependent transcriptional activation, chromatin remodeling, histone acetylation, histone acetyltransferase complexes, and histone binding, among others.

In the Australian subset, 33 GO terms and 2 KEGG pathways were significantly enriched (Fig. 4B and D; Supplementary Tables S5 and S7). In the GO enrichment analysis, the stress-related GO term “cytoplasmic stress granule” was significantly enriched in both the Australian and Ningxia subsets. Each subset contained six candidate genes under positive selection, two of which were shared between the subsets. Numerous RNA-related terms were significantly enriched, including rRNA maturation, ribosome assembly, and RNA surveillance and decay. The significantly enriched GO terms and KEGG pathways also included DNA unwinding, transcriptional elongation, translational elongation, histone and protein ubiquitination, histone acetyltransferase complex, redox metabolism, fatty acid biosynthesis, and cellular transport, among others.

Notably, among the positively selected candidate genes in the Ningxia and Australian subsets, seven and four genes, respectively, were annotated with the term “DNA repair” (GO:0006281; Supplementary Tables S4 and S5), but only one gene was shared between the two regional strain subsets. In the GO enrichment analysis of positively selected candidate genes from the Ningxia subset, “DNA repair” showed marginal statistical significance (*p* = 0.071 by the classic Fisher’s exact test; *p* = 0.049 by the weight01-weighted Fisher’s exact test). Two classes of histone acetyltransferase (HAT) complexes involved in DNA repair showed significant or marginally significant enrichment in both regional subsets (Supplementary Tables S4 and S5). One class was the NuA4 HAT complex (GO:0035267; *p*-values ranging from < 0.001 to 0.021); the other comprised the SAGA complex (GO:0000124) and the SLIK (SAGA-like) complex (GO:0046695; *p*-values ranging from 0.045 to 0.079). The positively selected candidate genes annotated with the term “SAGA complex” were identical to those annotated with the term “SLIK (SAGA-like) complex” in each regional strain subset.

### de novo assembly and annotation of *H. uvarum*

De novo assembly was performed on 148 *H. uvarum* strains, including 65 newly sequenced strains and 83 strains with unpublished genome assemblies (Supplementary Table S9). Their nuclear genome sizes and BUSCO completeness scores ranged from 8.68 to 8.92 Mbp (median: 8.74 Mbp) and from 80.02 to 81.05% (median: 80.77%), respectively. Both the assembly sizes and the BUSCO completeness scores met expectations. Although only second-generation sequencing data were used for de novo assembly in this study, the BUSCO completeness scores of these assemblies were comparable to or even slightly higher than those of the nearly complete genome of AWRI5759_A6, which was constructed using a combination of second- and third-generation sequencing data and achieved a BUSCO completeness score of 80.67%. This indicated that the newly assembled genomes achieved high assembly quality. In addition, 11 high-quality genome assemblies from public databases were selected for gene annotation (Supplementary Table S8). Their nuclear genome sizes and BUSCO completeness scores ranged from 8.09 to 9.10 Mbp (median: 8.87 Mbp) and from 77.40 to 80.91% (median: 80.67%), respectively.

Among the 148 nuclear genome assemblies generated in this study, annotated mRNA counts ranged from 4,136 to 4,253 (median: 4,174); among the 11 downloaded assemblies, they ranged from 3,916 to 4,364 (median: 4,181).

### Pangenomic analysis of *H. uvarum*

**Fig. 5:**
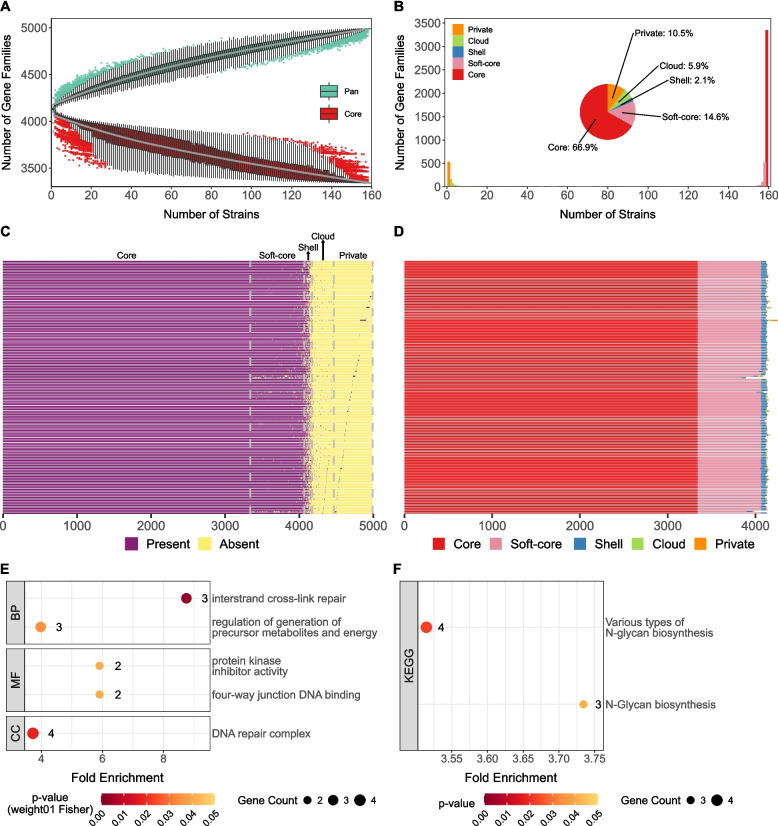
Pan-genome analysis of *H. uvarum*. **A** The pan-genome accumulation curve and core genome decline curve of 159 *H. uvarum* genomes; **B** Frequency distribution of the pan-genome. The histogram shows the number of gene families at different frequencies across the 159 *H. uvarum* genomes. The pie chart illustrates the proportion of gene families in each pan-genome category; **C** Presence-absence patterns of gene families across the 159 *H. uvarum* genomes; **D** Distribution of pan-genome categories across the 159 *H. uvarum* genomes. **E**, **F** GO and KEGG enrichment analyses of cloud and private genes. The numbers to the right of the dots represent the number of genes associated with the corresponding terms. The top 5 significant GO terms with *p* ≤ 0.05 for both the weight01 and classic Fisher methods are shown in subfigure E. Significant enrichment of KEGG pathways is shown in subfigure F. See Tables S10 and S11 for additional information. BP: Biological Process; MF: Molecular Function; CC: Cellular Component; KEGG: Kyoto Encyclopedia of Genes and Genomes

To capture the gene repertoire across all strains and systematically characterize presence-absence variation (PAV) in *H. uvarum*, we constructed a pangenome for this species. In addition to the 151 strains analyzed in the population genomics analysis, the pangenome analysis included 8 genome assemblies from public databases. Among the 159 strains included in the pangenome analysis, 4,991 gene families were identified. As more genomes were added, the number of gene families increased (Fig. 5A). The 3,340 gene families present in all 159 strains were defined as core gene families. The 728 gene families present in 144 to 158 strains (≥ 90% of the strains) were defined as soft-core gene families. The 107 gene families present in 16 to 143 strains (10%–90% of the strains) were defined as shell gene families. The 293 gene families present in 2 to 15 strains (≤ 10% of the strains) were defined as cloud gene families. The 523 gene families present exclusively in a single strain were defined as private gene families (Fig. 5B). Core, soft-core, shell, cloud, and private gene families accounted for an average of 80.91%, 17.40%, 1.46%, 0.15%, and 0.08% of the genes in a single strain, respectively (Fig. 5C–D). The pangenome of *H. uvarum* was determined to be open based on Heaps' Law analysis, which yielded a decay parameter α = 0.51 (*α* < 1 indicates an open pangenome). That is, the number of gene families continued to increase as more genomes were added, without approaching stabilization.

Enrichment analysis was performed on the combined set of cloud and private gene families, identifying 11 significantly enriched GO terms (*p* ≤ 0.05 in both the classic and weight01-weighted Fisher’s exact tests) and 2 KEGG pathways (*p* ≤ 0.05) (Fig. 5E–F; Supplementary Tables S10 and S11). Regarding metabolism, the significantly enriched GO terms and KEGG pathways included N-glycan biosynthesis, regulation of generation of precursor metabolites and energy, and tetrahydrofolate biosynthesis. Enriched terms related to nucleic acid metabolism and gene expression included DNA repair, DNA replication, transcription, and spliceosome assembly. Regarding development, the negative regulation of sporulation was significantly enriched. Notably, eight cloud and private gene families were annotated with the GO term “DNA repair” (GO:0006281), whose child term “DNA repair complex” (GO:1990391) was significantly enriched (Supplementary Table S10) and included four gene families.

## Discussion

Albertin et al. (2016) reported, based on microsatellite data, that *H. uvarum* strains isolated from France and South Africa formed a well-defined cluster according to geographical origin. Here, we analysed sequencing data from 151 strains sampled in China, Australia, and other continents. Phylogenetic analysis, ancestry coefficient analysis, and PCA generally distinguished Chinese strains from those sampled on other continents, further confirming the role of geographical isolation in driving intercontinental population divergence in *H. uvarum*. TreeMix analysis and *f*-branch statistics revealed substantial post-divergence gene flow and introgression between intercontinentally paired clades, suggesting ongoing intercontinental dispersal following geographic differentiation. A similar pattern has been observed across geographic regions and ecological niches in *S. cerevisiae*, driven by human activities or insect-mediated dispersal. For example, strains associated with coffee and cocoa have been found to migrate across multiple continents (Ludlow et al. 2016), and gene flow has also been reported across different fermentation environments, as well as between domesticated niches and wild ecological niches (Lee et al. 2022). Introgression has been observed between the Pacific West Coast Wine clade and other clades, such as the Transpacific Oak clade and the Wine/European clade (Marr et al. 2023).

Most candidate genes under positive selection may be region-specific. The candidate genes in the Ningxia subset and those in the Australian subset exhibited distinct GO term and KEGG pathway enrichment profiles. These findings suggested that environmental heterogeneity between the two regions had imposed divergent selection pressures on *H. uvarum*. In addition to revealing the pattern of positive selection through whole-genome scanning, our pangenome analysis also uncovered genomic features underlying the ecological adaptability of *H. uvarum*. A previous pangenome analysis of *H. uvarum*, based on only eight strains, indicated that its pangenome was closed and estimated that it contained fewer than 4,500 gene families (Guaragnella et al. 2020). In contrast, our analysis of 159 strains identified nearly 5,000 gene families and reclassified the *H. uvarum* pangenome as open. Notably, this open pangenome characteristic was observed even though our analysis focused on anthropogenic environments, a relatively homogeneous niche. Wild strains were not included in the analysis because the associated genomic data were not publicly available at the time this article was written. Strains harboring cloud or private gene families are likely to employ distinct adaptive mechanisms. This characteristic may confer broad ecological adaptability on *H. uvarum* across diverse environments.

In the GO enrichment analysis of positively selected candidate genes, multiple terms related to the positive regulation of filamentous growth in response to external stimuli were significantly enriched in the Ningxia subset. This functional profile aligned with the discussion of González et al. (2018), who inferred that the ability of *H. uvarum* to form pseudohyphae in rich media may confer an advantage for fruit colonization. These positively selected candidate genes may contribute to the ability of Ningxia strains to colonize fruit.

The GO term “cytoplasmic stress granule” (GO:0010494) was significantly enriched among the positively selected candidate genes in both the Ningxia and Australian subsets. Although research on cytoplasmic stress granules (SGs) in *Hanseniaspora* is currently lacking, SGs have been widely reported in various yeasts, including *S. cerevisiae*, *Schizosaccharomyces pombe*, and *Candida albicans* (Grousl et al. 2022). Under conditions such as heat shock, oxidative stress, and nutrient deprivation, translationally inactive mRNAs are sequestered into SGs, enabling preferential translation of stress-response mRNAs and thereby promoting cell survival under these stress conditions (Grousl et al. 2022). In this study, each subset contained six SG-related, positively selected candidate genes. However, only two genes were shared between the two subsets, indicating that the two regional strains experienced distinct environmental pressures.

Interestingly, although the genus *Hanseniaspora* has lost 14 genes annotated with the GO term “DNA repair” (GO:0006281), an additional 33 such genes were specifically lost in the *Hanseniaspora* FEL, which includes *H. uvarum* (Steenwyk et al. 2019). However, in the GO enrichment analysis of positively selected candidate genes from the Ningxia subset, the term “DNA repair” showed marginal significance, with seven genes included (Supplementary Table S4). In the Australian subset, four positively selected candidate genes were annotated with the term “DNA repair” (Supplementary Table S5); however, only one of these candidate genes was shared between the two regional strain subsets. This result suggested that populations from different geographical regions may have evolved divergent DNA repair mechanisms. Two classes of histone acetyltransferase (HAT) complexes involved in DNA repair were enriched or marginally enriched in the two regional strain subsets. The NuA4 HAT complex (GO:0035267) is specifically recruited to sites of DNA double-strand break repair (Bird et al. 2002; Cheng et al. 2021). The SAGA complex (GO:0000124) deubiquitination module promotes efficient DNA repair (Ramachandran et al. 2016). Furthermore, the SAGA complex collaborates with the NuA4 complex to repair DNA double-strand breaks by homologous recombination (Cheng et al. 2021). The term “chromatin remodeling” (GO:0006338) was significantly enriched in the Ningxia subset, and this biological process is also involved in DNA repair. GO enrichment analysis of the combined cloud and private gene families revealed significant enrichment of the "DNA repair complex" (GO:1990391), a child term of "DNA repair" (Supplementary Table S10). This suggested that a subset of *H. uvarum* strains may have acquired genes involved in DNA repair; however, the mechanisms by which these genes contribute to DNA repair remain to be elucidated.

This study offers new insights into the evolutionary patterns of *H. uvarum*, providing a foundation for advancing population genomics research on non-*Saccharomyces* yeasts. Future population genomics studies should incorporate a more diverse set of *H. uvarum* strains collected from broader geographic regions, more diverse ecological niches, and longer temporal spans to refine our understanding of the species’ evolutionary dynamics.

## Conclusion

We conducted population genomic and pangenomic analyses of *H. uvarum* strains sampled across multiple continents, primarily from China and Australia. Multiple independent analytical approaches (phylogenetic analysis, ancestry coefficient analysis, PCA, and TreeMix-inferred population trees) broadly distinguished Chinese strains from those sampled on other continents. However, TreeMix analysis and *f*-branch statistics inferred post-divergence gene flow and introgression between intercontinentally paired clades. Candidate genes under positive selection exhibited distinct patterns and functional enrichment profiles in the Ningxia and Australian strains, reflecting divergent selection pressures imposed by environmental heterogeneity between the two regions. In Ningxia, candidate genes under positive selection may facilitate *H. uvarum* colonization of fruit by positively regulating filamentous growth in response to external stimuli. Candidate genes associated with cytoplasmic stress granules were enriched in strains from both regions, although the specific gene sets differed between the two regional subsets. Despite sampling primarily from artificial niches and the absence of wild-niche strains in our dataset, *H. uvarum* exhibited an open pangenome, suggesting broad adaptive potential. This work advances our understanding of the evolutionary patterns of *H. uvarum*, offers high-quality genomic resources, and establishes a foundation for future ecological and industrial research on this species.

## Materials and methods

### Samples collection, DNA extraction and whole-genome sequencing

Yeast strains were collected from vineyards across the major wine-producing regions in northwest China, including Shaanxi, Ningxia, Gansu, and Xinjiang (Supplementary Fig. S1 and Table S1). Strain isolation and identification were performed as previously described (Li et al. 2023), and 65 strains of *H. uvarum* were identified in total.

These strains were subjected to whole-genome sequencing by BGI Genomics (China). Total genomic DNA was extracted using the phenol-chloroform-isoamyl alcohol method. The extracted DNA was randomly fragmented and processed through end repair, 3' adenylation, and adapter ligation. The resulting DNA library products were subjected to PCR amplification and quality control. The library products that passed quality control were converted into single-stranded libraries. Through a circularization reaction system, the DNA library products became single-stranded circular molecules. Paired-end sequencing was conducted on the DNBSEQ-T7 or MGISEQ-2000 platforms (MGI Tech, Shenzhen, China), generating 150 bp paired-end reads. Furthermore, publicly available raw sequencing data for 86 *H. uvarum* strains were retrieved from the NCBI Sequence Read Archive (SRA). The population genomic analysis included a total of 151 *H. uvarum* strains (Supplementary Table S1).

### VCF file creation

The VCF file was generated as follows: Adapter sequences and low-quality bases were removed from the raw reads using fastp (RRID: SCR_016962, v0.23.2) (Chen 2023) with the following parameters: --cut_front_mean_quality 20 --cut_front_window_size 1 --cut_right_mean_quality 20 --cut_right_window_size 4 --cut_tail_mean_quality 20 --cut_tail_window_size 1 --length_required 40. Additionally, for the downloaded data, reads aligning with the phiX174 genome (RefSeq accession: NC_001422.1) were filtered out using bbmap (RRID: SCR_016965, v39.01). The quality-filtered reads from each strain were mapped to the *H. uvarum* AWRI5759_A6 reference genome (GenBank accession: GCA_050947715.1) using bwa-mem2 (RRID: SCR_022192) (Vasimuddin et al. 2019) with default parameters. The resulting BAM files were sorted by genomic coordinates, and duplicate reads were removed from the sorted BAM files using Samtools (RRID: SCR_002105, v1.17) (Danecek et al. 2021).

Germline short variants were called using the Genome Analysis Toolkit (GATK) Best Practices workflow (Van der Auwera and O'Connor 2020). The HaplotypeCaller module in GATK (RRID: SCR_001876, v4.3.0.0) was used to identify SNPs and InDels in nuclear DNA for each sample individually and to generate gVCF files. The gVCF files from all samples were jointly genotyped using the GenotypeGVCFs module in GATK (v4.3.0.0) to produce a joint VCF file. The joint VCF file was subjected to hard filtering based on the recommended parameters in the official GATK tutorial. The specific criteria were: QD < 2.0, QUAL < 30.0, SOR > 3.0, FS > 60.0, MQ < 40.0, MQRankSum < −12.5, and ReadPosRankSum < −8.0. Ultimately, this process produced a joint VCF file containing data from 151 samples.

Owing to the relatively short read lengths of second-generation sequencing, multiple alignments often occur in repetitive and low-complexity regions, leading to decreased accuracy in variant calling. To mitigate this issue, the project identified and masked repetitive and low-complexity regions in the reference genome AWRI5759_A6. Specifically, EDTA (RRID: SCR_022063, v2.0.1) (Ou et al. 2019) was used to generate a non-redundant transposable element library file (TElib) for the AWRI5759_A6 genome with the parameter “--sensitive 1”. This TElib file served as a custom library for RepeatMasker (RRID: SCR_012954, v4.1.5) to annotate repetitive and low-complexity genomic regions using the parameters “-s -norna -no_is”. Finally, SNPs within repetitive and low-complexity regions were filtered out from the VCF file using the SelectVariants module in GATK (v4.3.0.0).

### Population genomic analysis

#### Phylogenetic analysis

Phylogenetic analysis was conducted using two different analytical frameworks: (1) The maximum likelihood (ML) phylogenetic tree was inferred from the concatenated CDS alignment, with *Hanseniaspora nectarophila* designated as the outgroup. (2) A dissimilarity matrix was calculated from genome-wide SNP data for paired strains, and a BioNJ tree was inferred from this matrix and midpoint-rooted.

##### Construction of the ML tree

The genome assembly of *H. nectarophila* (GenBank accession: GCA_030573495.1) was annotated using funannotate (v1.8.5), as described in the “Annotation of the genome” section. The VCF file of *H. uvarum* was converted into a FASTA file, with heterozygous sites encoded using International Union of Pure and Applied Chemistry (IUPAC) ambiguity codes. OrthoFinder (RRID: SCR_017118, v2.5.5) (Emms and Kelly 2019) was used to identify orthogroups across all analyzed strains. FASMA (v2.4.1) was used to align the sequences within each orthogroup, and TrimAl (RRID: SCR_017334, v1.5.1) was used for alignment trimming. An unrooted phylogenetic tree of *H. uvarum* was inferred from the concatenated alignment of 4,134 one-to-one orthologous genes using IQ-TREE (RRID: SCR_017254, v3.0.1) with 1,000 ultrafast bootstrap replicates, using the following parameters: `-s $Concatenated.cds.without_outgroup.fa -m MFP -mset GTR -B 1000 -T $threads -seed 1000 -pre $ingroup`. This unrooted tree was subsequently used as a topological constraint to infer the final rooted tree in IQ-TREE, with *H. nectarophila* designated as the outgroup. The rooted tree was inferred from the concatenated alignment of 2,404 one-to-one orthologous genes shared between *H. uvarum* and *H. nectarophila*, using the following parameters: `-s $Concatenated.cds.with_outgroup.fa -g ingroup.treefile -m MFP -mset GTR -o H.nectarophila -T $threads -seed 1000 -pre $final.ML`.

##### Construction of the BioNJ tree

The VCF file was converted into a GDS file using the `snpgdsVCF2GDS` function from the R package SNPRelate (RRID: SCR_022719, v1.28) (Zheng et al. 2012). A dissimilarity matrix was calculated for pairwise comparisons among strains using the `snpgdsDiss` function from the R package SNPRelate, based on 432,573 genome-wide biallelic SNPs. The BioNJ tree was inferred from this matrix using the BioNJ algorithm, as implemented in the R package ape (RRID: SCR_017343, v5.8-1) (Paradis and Schliep 2019). Jackknife resampling was performed with 1,000 replicates and a removal probability of ${e}^{-1} (\approx 36.79\mathrm{\%})$ (Farris et al. 1996). The tree was rooted using the midpoint rooting method implemented in the R package phytools (RRID:SCR_015502, v2.5-2), and was visualized using ggtree (RRID: SCR_018560, v3.2.1) (Yu et al. 2018).

#### Ancestry coefficient analysis

sNMF (v1.2) (Frichot et al. 2014) was used to estimate the ancestry coefficient. Unlike commonly used methods such as STRUCTURE (Falush et al. 2003) and ADMIXTURE (Alexander et al. 2009), sNMF does not rely on the assumption of unrelated individuals, making it suitable for analyzing inbred populations. Linkage disequilibrium pruning was performed using PLINK (RRID: SCR_001757, v1.9) with the following parameters: “--indep-pairwise 50 10 0.1 --geno 0.2”. Next, sNMF (v1.2) was run with *K* values ranging from 1 to 30. For each *K* value, the algorithm was executed 10 times with different fixed seed initialization values. The run with the lowest cross-entropy value for each *K* value was selected as the final result. The *K* value corresponding to the minimum cross-entropy was selected as the optimal *K*.

#### Principal component analysis

Principal component analysis (PCA) was performed using smartpca (v18140) (Patterson et al. 2006; Price et al. 2006) within EIGENSOFT (RRID: SCR_004965, v8.0.0). The PCA results were visualized using R.

#### Population differentiation and nucleotide diversity

Genome-wide average nucleotide diversity (π) and average nucleotide divergence (*d*_*XY*_) were calculated using pixy (Korunes and Samuk 2021). Hudson's *F*_ST_ values (Hudson et al. 1992; Bhatia et al. 2013) between population pairs were calculated using PLINK (v2.0) (Chang et al. 2015).

Nucleotide diversities (π) of the Ningxia subset and the Australian subset, as well as Hudson's *F*_ST_ between them, were calculated using Pixy with a window size of 1000 bp and a step size of 50 bp. When a gene’s CDS region overlapped with multiple analysis windows, only the windows with the longest overlap were retained, including all windows that shared the same maximum overlap length.

#### Gene flow and introgression

The gene flow between paired clades was analyzed using TreeMix (RRID: SCR_021636) (Pickrell and Pritchard 2012). Parameters were set to “-bootstrap -global -k 1000”, with the USA clade designated as the outgroup. This analysis was repeated 10 times for each *m* value using different seed initialization values. The `linear` function in OptM (v0.1.6) (Fitak 2021) was used to estimate the optimal number of TreeMix migration edges. For each value of *m*, the run with the highest log-likelihood was retained as the final result.

Estimation of introgression between paired clades was performed using the *f*-branch statistic (*f*_*b*_) (Malinsky et al. 2018), an extension of the *f*4-ratio statistic, as implemented in Dsuite (Malinsky et al. 2021), with the USA clade designated as the outgroup. In the calculation of the *f*_*b*_ statistic, the AUS-I and AUS-II clades were excluded because they cluster with USA to form a monophyletic group in the ML tree or BioNJ tree.

Prior to the analyses, strains exhibiting discordant clustering between the ML and BioNJ trees were excluded: YC-C-1, JY-C-2-3, JY-D-2-1, AWRI5759_D4, and AWRI5759_B2 (Supplementary Fig. S2). Three putative “migrant” strains were also excluded, for example, strain AWRI1185, which was assigned to the AUS-IX clade in the phylogenetic analysis but was isolated in the USA. Details on strain inclusion and exclusion in the TreeMix analysis and* f*_*b*_ statistics are provided in Supplementary Table S1.

#### Positive selection analysis

RAiSD (v2.9) (Alachiotis and Pavlidis 2018) was used to identify candidate genes under positive selection based on the Ningxia (China) subset and the Australian subset, respectively. Prior to analysis, clonal strains in the two subsets were identified and removed using the KING-Robust kinship estimator (Manichaikul et al. 2010) in PLINK 2. RAiSD constructs a comprehensive statistic μ to detect positively selected regions by integrating the site frequency spectrum (SFS), linkage disequilibrium (LD) patterns, and genetic diversity. Sites detected by RAiSD with μ values in the top 0.5% were designated as candidate sites under positive selection. GO enrichment analysis was conducted using topGO (RRID: SCR_014798, v2.60.1) on genes containing these candidate sites. *P*-values were calculated using two methods: the weight01 Fisher test and the classic Fisher test. GO terms were considered significant only if *p*-values from both methods were less than 0.05. KEGG pathway enrichment analysis was performed using clusterProfiler (RRID: SCR_016884, v4.18.2) (Wu et al. 2021), and pathways with a *p*-value less than 0.05 were considered significant.

The GO (RRID: SCR_002811) and KEGG (RRID: SCR_012773) annotations of the reference genome AWRI5759_A6 were obtained from eggNOG-mapper (RRID: SCR_021165) (Cantalapiedra et al. 2021) using default parameters (accessed on November 16, 2025).

### De novo assembly and annotation of the genomes

#### De novo assembly of the genomes

De novo assembly was performed on 65 strains with newly sequenced data from this study and 83 strains with unpublished genome assemblies (Supplementary Table S9). The genomes were assembled using SPAdes (RRID: SCR_000131, v4.0.0) (Prjibelski et al. 2020). Initial assembly employed default k-mer values. If the assembled genome size was abnormal, k-mer parameters were reset to “-k 21, 33, 55, 67” and iteratively adjusted in 2-step increments until reaching 89. Redundans (v2.0.1) (Pryszcz and Gabaldón 2016) was used to remove redundant sequences from the initially assembled contigs, construct scaffolds, and close gaps in the assemblies. Using RagTag (RRID: SCR_027293, v2.1.0) (Alonge et al. 2022), “pseudo-chromosome” assemblies for each sample were generated by connecting scaffolds based on the reference genome (*H. uvarum* AWRI5759_A6, GenBank accession: GCA_050947715.1). The assemblies were polished using NextPolish (RRID: SCR_025232, v1.4.1) (Hu et al. 2019). Contamination in the assemblies was removed using FCS-GX (RRID: SCR_026367, v0.4.0) (Astashyn et al. 2024). BASTA (v1.4.1) (Kahlke and Ralph 2019) was used to filter out scaffolds matching the mitochondrial DNA of *H. uvarum* (GenBank accession: CM114679.1). Finally, BUSCO (RRID: SCR_015008, v5.4.7) (Manni et al. 2021) and QUAST (RRID: SCR_001228, v5.2.0) (Gurevich et al. 2013) were used to evaluate the final assembled genomes.

Additionally, 19 assembled genomes from 16 strains were retrieved from the NCBI database (accessed on October 1, 2025). Using the same method as described in the previous paragraph, “pseudo-chromosome” assemblies were constructed by connecting their scaffolds, and assembly contamination was detected and removed. After evaluating pseudochromosome quality and counting the number of annotated genes (the annotation method is described in the next subsection), we selected 11 high-quality strain assemblies for pangenome analysis. Each assembly contained a reasonable number of annotated genes (Supplementary Tables S8 and S9). Among these 11 strains, 8 were not included in the aforementioned population genomics analysis because the WGS raw data were unavailable for them. As a result, these 8 strains could not undergo the same variant calling pipeline as that used for the available WGS raw reads, which prevented uniform variant calling quality control. This may compromise the reliability of the results of the population genomics analysis.

#### Annotation of the genome

Although some genomes have been publicly annotated, to avoid bias caused by different annotation pipelines, this study adopted a unified pipeline to annotate all genomes. Gene prediction and annotation of the assembled genomes were performed using funannotate (RRID: SCR_023039, v1.8.5). First, repetitive and low-complexity regions in each assembled genome were soft-masked using the method described in the “VCF file creation” subsection. Second, gene prediction was conducted on the soft-masked assemblies using the gene prediction module in funannotate (v1.8.5). In the gene prediction process, results from two methods were combined: Augustus (RRID: SCR_008417, v3.5.0) (Stanke et al. 2008) and GeneMark-ES (RRID: SCR_011930, v4.71) (Ter-Hovhannisyan et al. 2008). The training of gene prediction models was based on publicly available RNA data from QTX-C10 (BioProject in NCBI: PRJNA1110608). Protein evidence was derived from UniProtKB/Swiss-Prot (v2023_05). Third, the gene annotation module was used to annotate the genes. The databases employed for annotation included InterProScan (RRID: SCR_005829, v5.61-93.0) (Paysan-Lafosse et al. 2022), antiSMASH (RRID: SCR_022060, v7.1.0) (Blin et al. 2023), emapperdb (v5.0.2) (Cantalapiedra et al. 2021), Pfam (v36.0) (Mistry et al. 2020), UniProtKB/Swiss-Prot (v2023_05) (The UniProt Consortium 2022), MEROPS (RRID: SCR_007777, v12.0) (Rawlings et al. 2017), and dbCAN2 (v12.0) (Zhang et al. 2018).

### Pangenome analysis

Based on the nucleotide sequence FASTA files of the predicted genes, OrthoFinder (RRID: SCR_017118, v2.5.5) (Emms and Kelly 2019) was run to determine the gene families. Based on the analysis results from OrthoFinder, pangenomic statistics were performed using custom R scripts. In this study, gene families were classified into five categories based on their prevalence across strains: core gene families (present in all strains), soft-core gene families (present in > 90% of strains), shell gene families (present in 10%–90% of strains), cloud gene families (present in < 10% of strains), and private gene families (present exclusively in a single strain).

The heaps function in the R package micropan was employed to assess whether pangenome gene families were open or closed. The heaps function is based on Heaps' Law and is calculated as follows (Tettelin et al. 2008):

$$\begin{array}{c}n=\kappa {N}^{-\alpha }\end{array}$$

Where *N* is the number of newly added sequenced genomes, *n* is the corresponding number of newly added genes, and *α* is the decay parameter, a pangenome is considered closed when *α* > 1.0 and open when *α* < 1.0.

Use the method described in the subsection “Positive selection analysis” to perform GO (RRID: SCR_002811) and KEGG (RRID: SCR_012773) pathway enrichment analyses on the combined set of the cloud gene family and the private gene family.

## Supplementary Information

Supplementary Material 1: Supplementary Figures S1-S8.

Supplementary Material 2: Supplementary Tables S1-S11.

Supplementary Material 3: Supplementary Note.

## Data Availability

New sequence data were deposited in the National Center for Biotechnology Information (NCBI) under BioProject accession number PRJNA1199399.

## Abbreviations

FEL: The Faster-Evolving Lineage (of *Hanseniaspora*)
GO: Gene Ontology
HAT: Histone acetyltransferase
KEGG: Kyoto Encyclopedia of Genes and Genomes
PCA: Principal Component Analysis
SEL: The Slower-Evolving Lineage (of *Hanseniaspora*)
SG: Stress granule
WGS: Whole-genome sequencing
