# Supplementary material for: Population genomics reveals gene flow and positive selection patterns in the wine-related yeast Hanseniaspora uvarum
Source: Stress Biol. 2026 Jul 27;6(1):54. doi: 10.1007/s44154-026-00319-z (PMC13407411; doi:10.1007/s44154-026-00319-z)
Supplement: Supplementary file 1 — Supplementary Material 1: Supplementary Figures S1-S8. [file 44154_2026_319_MOESM1_ESM.pdf]

**Supplementary Figure S1**

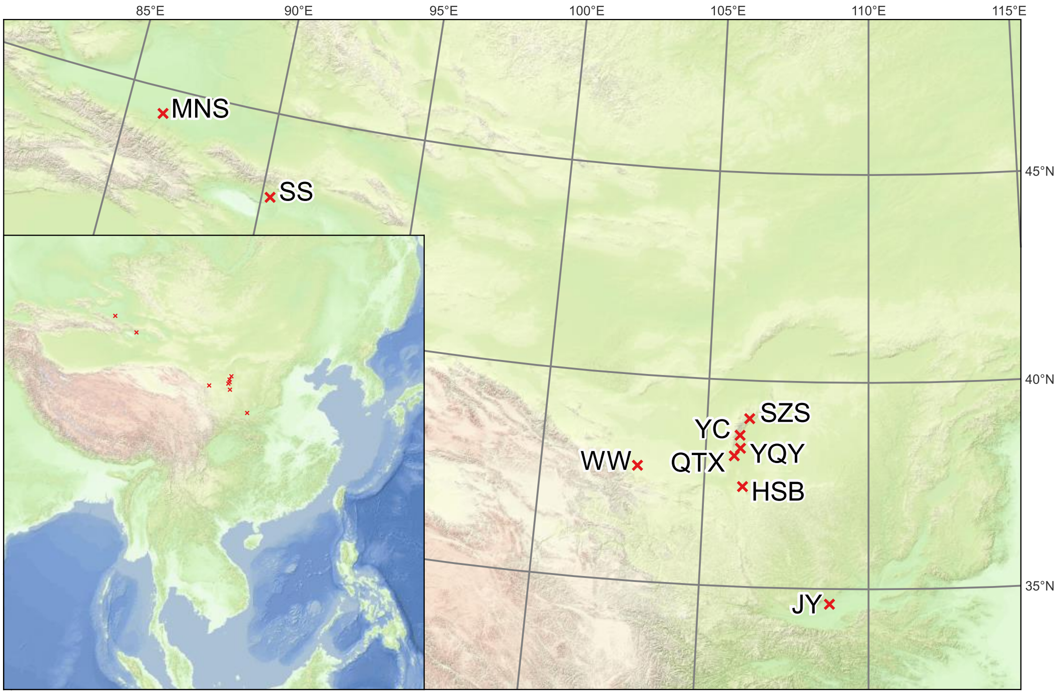


**Fig. S1** Sampling Locations of Isolated Strains in Northwest China

The Meanings of English Abbreviations in Maps:

**JY:** Jingyang County, Shaanxi Province;

**SZS:** Shizuishan City, Ningxia Hui Autonomous Region;

**YC:** Yinchuan City, Ningxia Hui Autonomous Region;

**YQY:** Yuquanying and the adjacent Minning Town, Yongning County, Ningxia Hui Autonomous Region;

**QTX:** Qingtongxia City, Ningxia Hui Autonomous Region;

**HSB:** Hongsibu District, Wuzhong City, Ningxia Hui Autonomous Region;

**WW:** Wuwei City, Gansu Province;

**SS:** Shanshan County, Xinjiang Uygur Autonomous Region;

**MNS:** Manas County, Xinjiang Uygur Autonomous Region

## Supplementary Figure S2

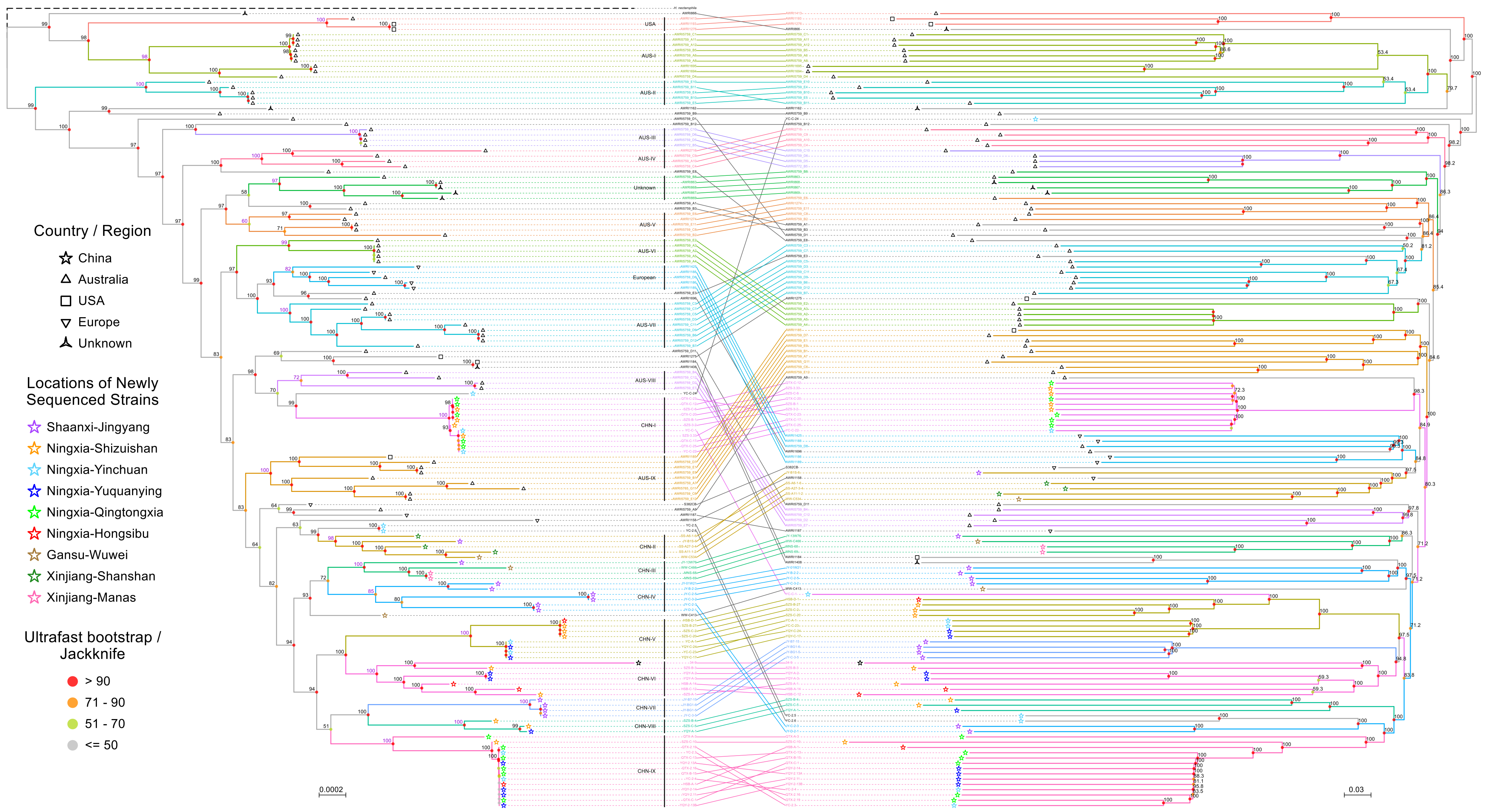


**Fig. S2** Comparison of the ML and BioNJ trees

**Left:** The ML tree was inferred from a concatenated alignment of one-to-one orthologous genes, with *H. nectarophila* designated as the outgroup. Ultrafast bootstrap support values are shown at the nodes. **Right:** The BioNJ tree was inferred from a genome-wide SNP-based dissimilarity matrix and was midpoint-rooted (no outgroup was used). Jackknife support values (removal probability = $e^{-1} \approx 36.79\%$) are shown at the nodes.

## Supplementary Figure S3

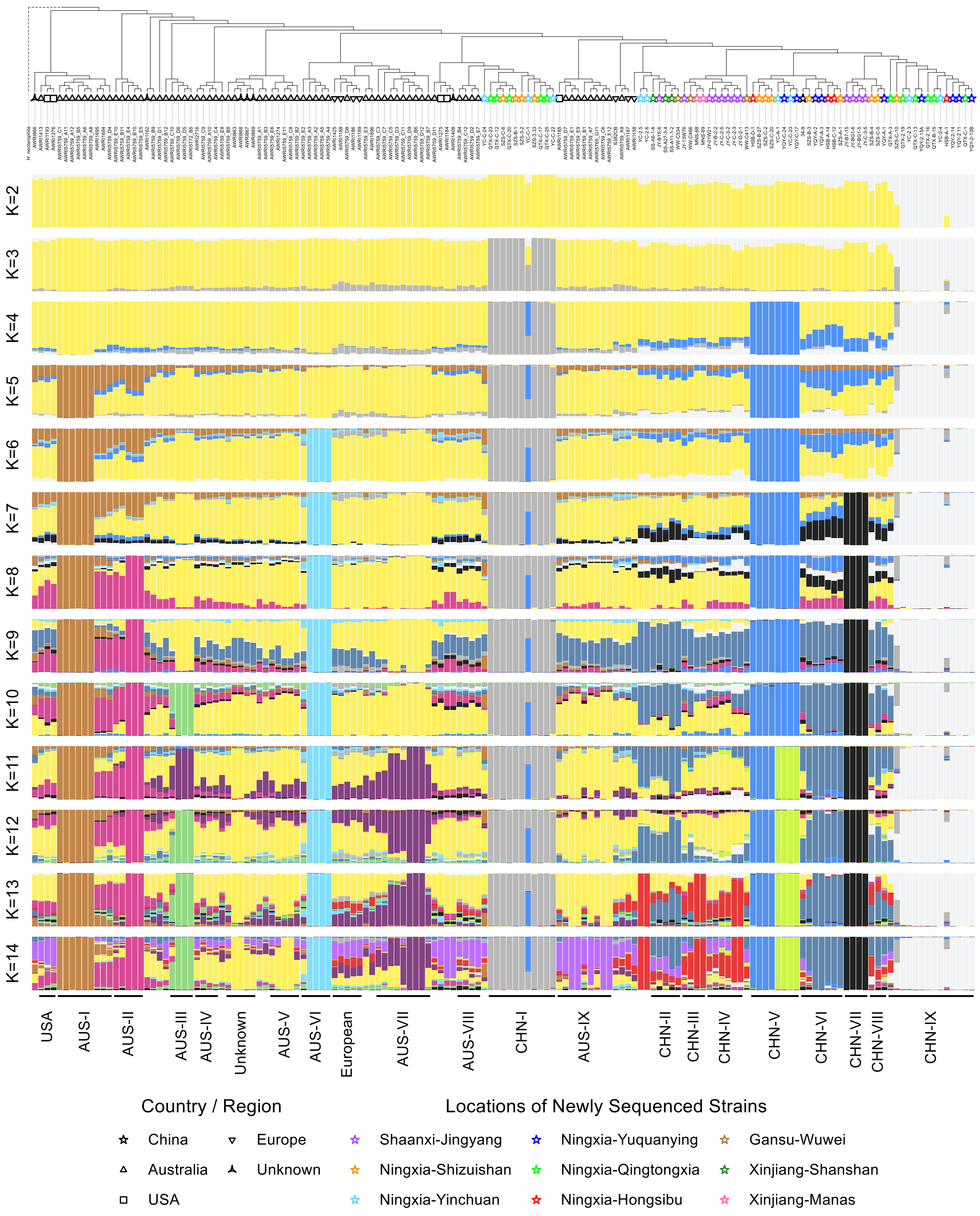


**Fig. S3** Ancestry coefficient analysis (K = 2–14)

# Supplementary Figure S4

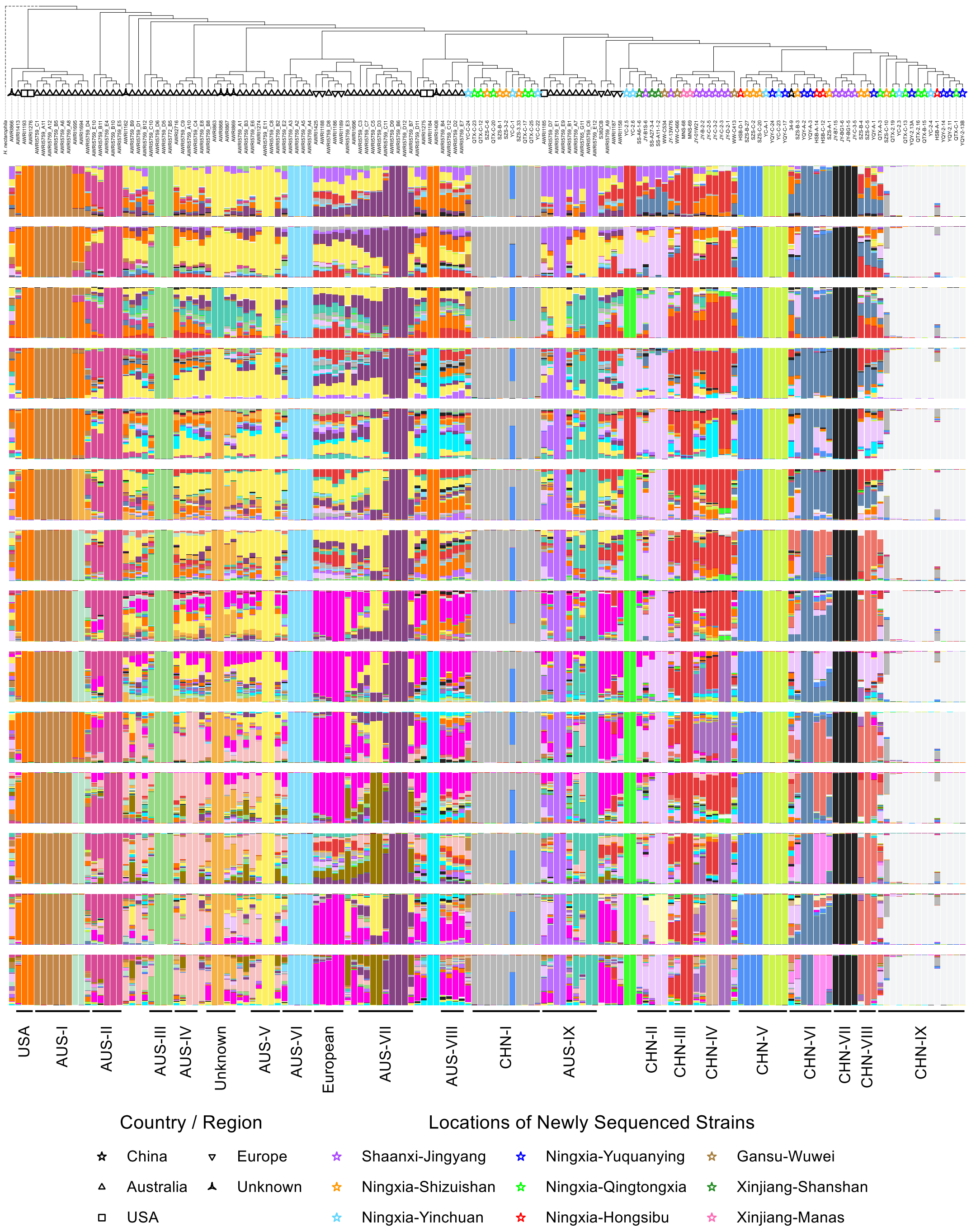


**Fig. S4** Ancestry coefficient analysis (K = 15–28)

**Supplementary Figure S5**

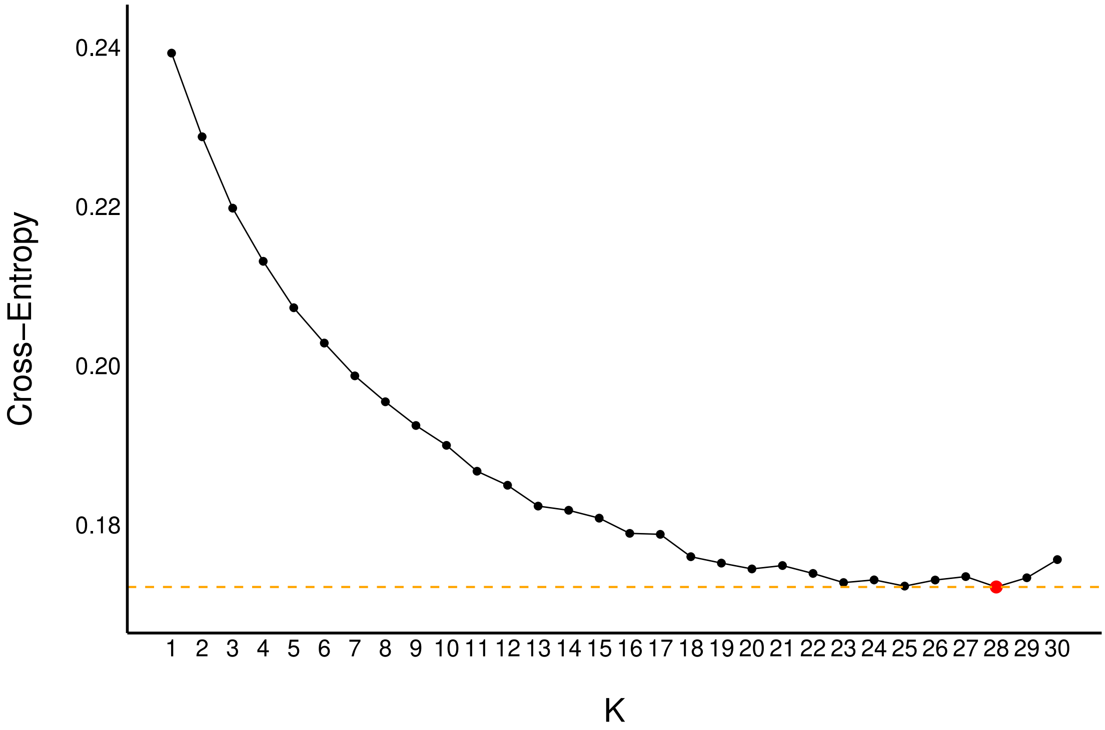


**Fig. S5** Cross−Entropy of sNMF

**Supplementary Figure S6**

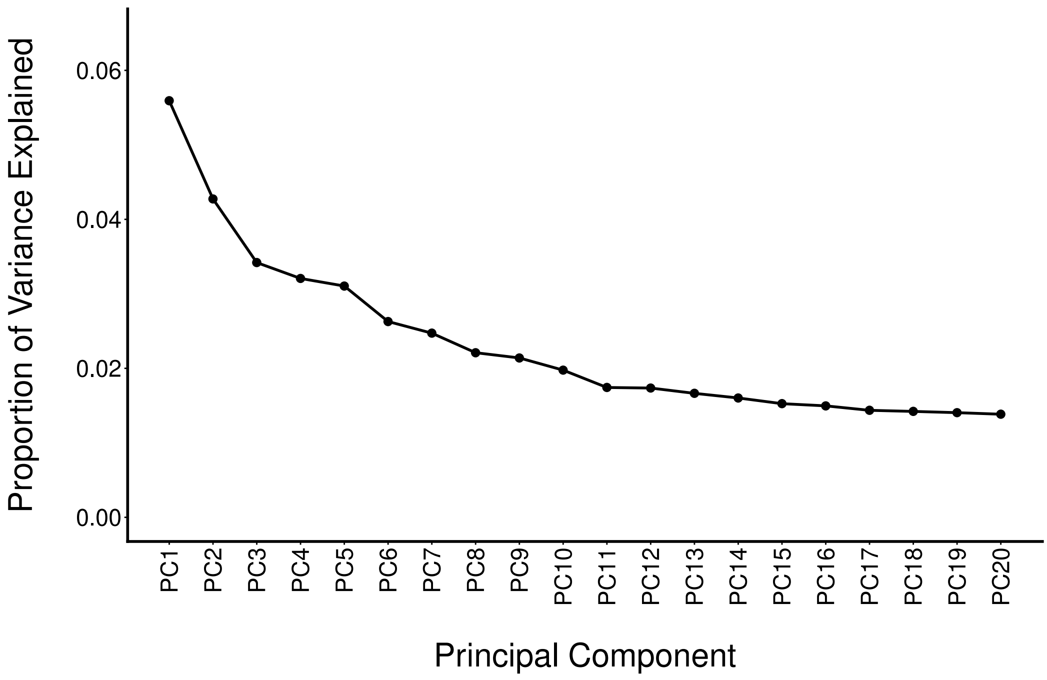


**Fig. S6** Proportion of variance explained by each principal component

# Supplementary Figure S7

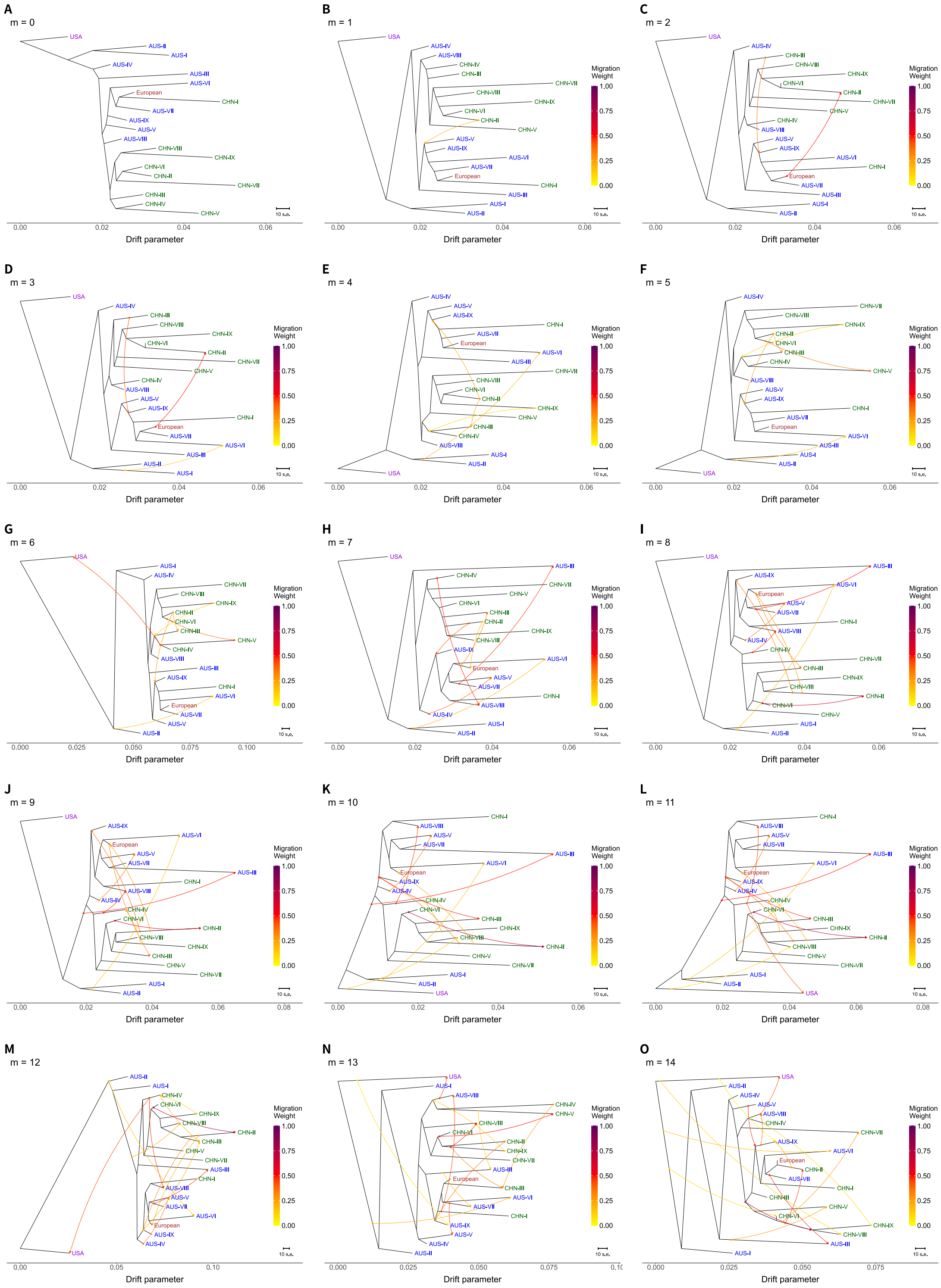

**Fig. S7** Treemix results between paired clases for *H. uvarum*

**A–O** Migration edges (*m* values) cover 0–14

**Supplementary Figure S8**

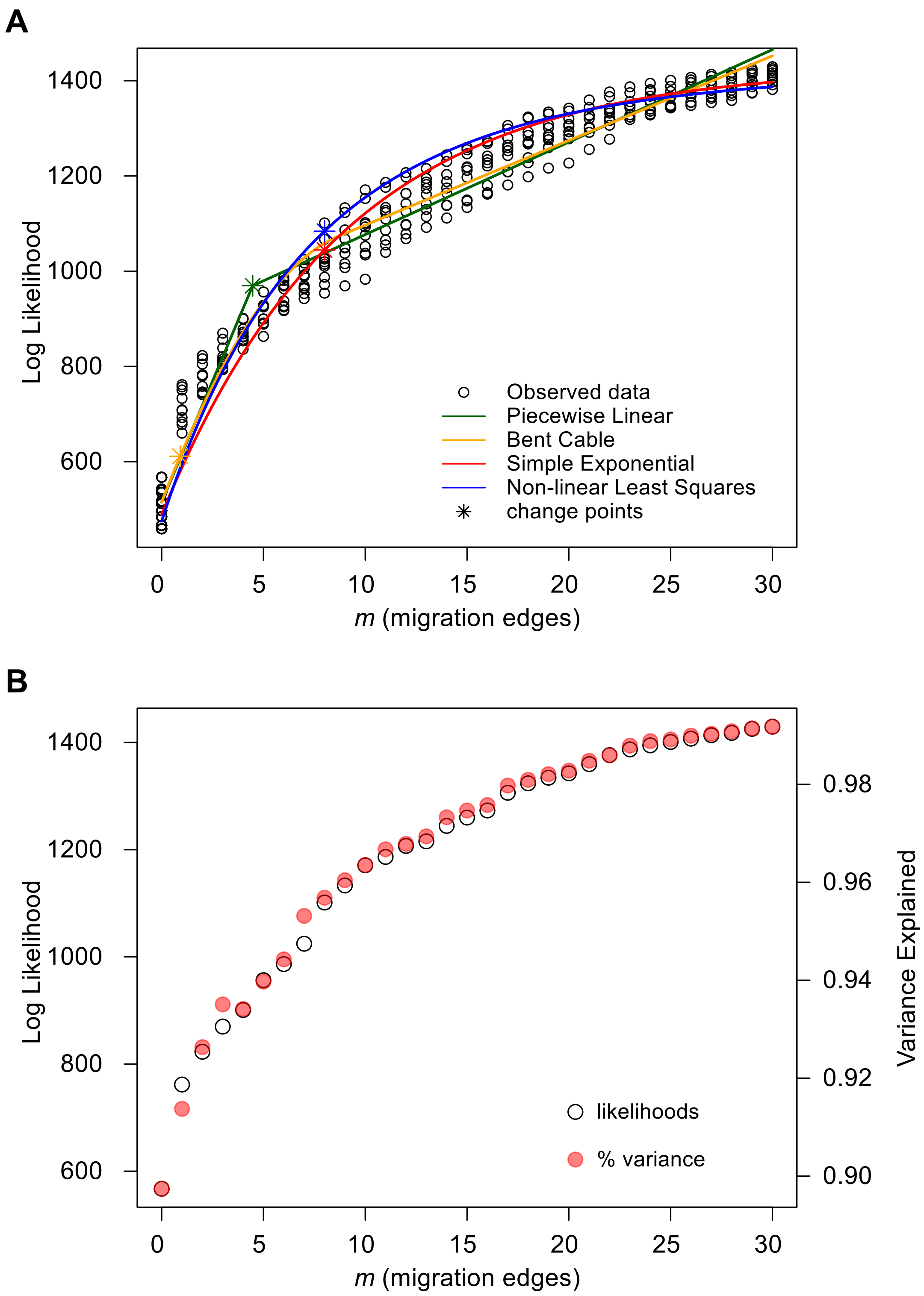


**Fig. S8** Estimating the optimal number of migration edges using OptM

**A** Estimating the optimal number of migration edges using four parametric models **B** Log-likelihood and the proportion of variance explained for each migration edge
