# Supplementary material for: Population genomics reveals gene flow and positive selection patterns in the wine-related yeast Hanseniaspora uvarum
Source: Stress Biol. 2026 Jul 27;6(1):54. doi: 10.1007/s44154-026-00319-z (PMC13407411; doi:10.1007/s44154-026-00319-z)
Supplement: Supplementary file 3 — Supplementary Material 3: Supplementary Note. [file 44154_2026_319_MOESM3_ESM.pdf]

Supplementary Note

Additional details on phylogenetic tree

We delimited clades based on phylogenetic evidence from both the ML and BioNJ tree topologies, as well as on geographic sampling information. When phylogenetic trees are inferred using different analytical workflows or algorithms, their topologies and clustering patterns often differ. However, the ML and BioNJ trees yielded largely consistent clade assignments. In the ML tree, 129 of the 151 strains (85.4%) were assigned to 21 clades. Among these 129 strains, only five exhibited inconsistent clade assignments between the two trees (Supplementary Fig. S2). Specifically:

(1) Strain YC-C-1 was assigned to clade CHN-I in the ML tree but to clade CHN-V in the BioNJ tree. Ancestry coefficient analysis indicated that this strain harbors genetic signatures characteristic of both clades CHN-I and CHN-V (Figure 1B; Supplementary Figs. S3–S4).

(2) All six strains in the CHN-IV clade originated from Jingyang County, China, and formed a monophyletic group in the ML tree. The BioNJ tree revealed a topological difference: two of these Jingyang strains (JY-C-2-3 and JY-D-2-1) clustered with two strains from Yinchuan City, China (YC-2.5 and YC-2.6). However, the remaining four strains in the CHN-IV clade consistently clustered together in both phylogenetic trees. We therefore retained the CHN-IV designation based on the monophyly of all six strains in the ML tree, their shared geographic origin, and the consistent clustering of the majority (4/6) of isolates across both analytical methods.

(3) Strain AWRI5759_D4 was assigned to clade AUS-I in the ML tree but to clade AUS-II in the BioNJ tree; AUS-I and AUS-II were sister clades in the BioNJ tree.

(4) Strain AWRI5759_B2 was assigned to clade AUS-V in the ML tree. In the BioNJ tree, this strain, along with clade AUS-VII and four additional strains (AWRI5759_A1, AWRI5759_B3, AWRI5759_D1, and AWRI5759_E8), formed the sister group to clade AUS-V.

The above five strains were excluded from the gene flow and introgression analyses (i.e., the TreeMix analysis and f-branch statistic calculations).

We performed the ML tree analysis with 1,000 ultrafast bootstrap replicates and the BioNJ tree analysis with 1,000 jackknife replicates (removal probability = $e^{-1} \approx$ 36.79%). In the ML tree, ultrafast bootstrap support values for 20 clades exceeded 70%,

and those for 17 clades exceeded 95% (Fig. 1A; Supplementary Fig. S2). The bootstrap support value for AUS-V, which comprised five strains, was the lowest (60%) in the ML tree. However, four of these strains formed a monophyletic clade with a jackknife support value of 100% in the BioNJ tree (Supplementary Fig. S2). As mentioned earlier, another strain (AWRI5759_B2) showed a different clustering pattern in the BioNJ tree.
